# Mismatch Repair (MMR) Gene Mutation Carriers Have Favorable Outcome in Colorectal and Endometrial Cancer: A Prospective Cohort Study

**DOI:** 10.3390/cancers16132342

**Published:** 2024-06-26

**Authors:** Jiunn-Tyng Yeh, Hung-Pin Peng, Fei-Hung Hung, Chen-Fang Hung, Ling-Ling Hsieh, An-Suei Yang, Yong Alison Wang

**Affiliations:** 1 Department of Internal Medicine, Koo Foundation Sun Yat-Sen Cancer Center, Taipei 11259, Taiwan; tyng1122@gmail.com (J.-T.Y.); llhsieh@kfsyscc.org (L.-L.H.); 2 Biomedical Translation Research Center, Academia Sinica, Taipei 11571, Taiwan; peng1234@gate.sinica.edu.tw; 3 Health Data Analytics and Statistics Center, Office of Data Science, Taipei Medical University, Taipei 11013, Taiwan; fionhung@tmu.edu.tw; 4 Department of Research, Koo Foundation Sun Yat-Sen Cancer Center, Taipei 11259, Taiwan; cfhorng@kfsyscc.org; 5 Genomics Research Center, Academia Sinica, Taipei 11571, Taiwan; yangas@gate.sinica.edu.tw; 6 National Yang Ming Chiao Tung University School of Medicine, Taipei 112304, Taiwan

**Keywords:** DNA mismatch repair, Lynch syndrome, colorectal cancer, endometrial cancer, high-throughput nucleotide sequencing

## Abstract

**Simple Summary:**

Germline and somatic deficiencies of mismatch repair proteins (MMRd) are related to colorectal and endometrial cancers, but the survival impact in Asian patients remains unclear. We investigated the prevalence and outcomes of patients with germline and/or somatic MMRd. Germline and somatic MMRd were determined by gene sequencing and protein staining of tumor samples, respectively. We found that colorectal and endometrial cancer patients with germline MMRd have favorable survival outcomes compared to those without, regardless of somatic MMRd status. These findings highlight the importance of germline genetic testing when tumor MMRd is detected.

**Abstract:**

Germline (Lynch syndrome, LS) and somatic deficiencies of mismatch repair proteins (MMRd) are linked to colorectal and endometrial cancer; however, their prognostic impact in Asian populations remains unclear. This prospective cohort study aimed to determine the prevalence and outcome of germline and somatic MMRd in cancer patients suspected of LS. Patients with colorectal or endometrial cancer suspected of LS were enrolled and underwent gene sequencing for germline MMRd (gMMRd) and immunohistochemistry staining of MMR proteins in a subset of the pathological samples (pMMRd). Among the 451 enrolled patients, 36 patients were gMMRd (+). Compared with gMMRd (−) patients, the 10-year relapse-free survival in gMMRd (+) patients was significantly higher (100% vs. 77.9%; *p* = 0.006), whereas the 10-year overall survival was similar (100% vs. 90.9%; *p* = 0.12). Among the 102 gMMRd (−) patients with available pMMR status, 13.7% were pMMRd (+). The 5-year relapse-free survival was 62.9% in gMMRd (−) pMMRd (+) patients and 35.0% in gMMRd (−) pMMRd (−) patients, both lower than gMMRd (+) patients (100%; *p* < 0.001). This study showed that having LS confers a favorable outcome in colorectal and endometrial cancer patients and highlights the importance of germline genetic testing following the detection of somatic MMRd.

## 1. Introduction

Lynch syndrome (LS) is the most common hereditary cancer syndrome, for which the oncogenicity results from heterozygous germline pathogenic variants in genes involved in the mismatch repair (MMR) mechanism, including *MLH1*, *MSH2*, *MSH6*, and *PMS2* [1]. DNA mismatches due to replication-associated errors or DNA damage (e.g., methylation, oxidation, or interstrand crosslink) causing base mismatch or single nucleotide insertion/deletion errors are recognized by the MSH2-MSH6 dimer (MutSα) [2]. The binding of MutSα to the DNA strand with mismatch leads to the recruitment of the MLH1-PMS2 dimer (MutLα) and initiates the DNA repair process. Pathogenic variants of the mismatch repair genes (also known as MMR deficiency, MMRd) cause high instability of the microsatellite regions of the genome, which are prone to mismatch errors. The accumulation of mutations in the microsatellite regions and elsewhere in the genome results in tumorigenesis. Lynch syndrome is known as the most common cause of heritable colorectal cancer (CRC), and the only heritable cause of endometrial cancer (EC) [3,4], accounting for about 2.1% of all cases of endometrial carcinoma and 2.2% of colorectal adenocarcinoma [5,6].

LS-associated cancers have diverse pathologic and clinical features and differ from non-LS-associated neoplasms in pathology, clinical characteristics, and outcomes [7]. Multi-gene cancer panel tests have been employed in suspected LS patients to identify the genotype, leading to increased diagnostic accuracy and more precise management as the risks of cancer for each genotype are well described [8,9,10]. Studies have shown that LS-associated CRCs have a better prognosis than CRCs with intact mismatch repair functionality [11,12,13,14,15,16,17]. However, long-term outcome data of LS patients with CRC and EC are lacking, especially for the Asian population.

With the increasing utilization of somatic gene testing and immunohistochemistry (IHC) for cancer patients suspected of LS, a subset of patients with microsatellite instability (MSI) or loss of MMR protein expression by IHC in the tumor tissue but without germline MMR pathogenic variants could be identified. This population is heterogeneous, encompassing special cases of Lynch syndrome resulting from hypermethylation of the *MLH1* promoter BRAF V600E somatic mutation that is considered sporadic cancer, and “Lynch-like syndrome (LLS)” if the previous two alterations are absent [18,19]. Due to the diverse etiologies and the fact that MSI or MMR IHC in tumors are not routinely tested, the prevalence and outcomes of patients with only tumor pathological MMR deficiency (pMMRd) without germline MMR deficiency (gMMRd) are not well-understood.

This study aims to investigate the prevalence and long-term outcomes of germline MMR deficiency (gMMRd) in a Chinese cohort of cancer patients with suspected LS. We further identified the gMMRd (−) pMMRd (+) subgroup by retrospectively reviewing tumor IHC staining and compared their long-term outcomes to patients with sporadic cancer and to those with germline MMR deficiency. The insights gained from this study will contribute to a deeper understanding for the interplay between tumorigenesis and MMRd, as well as surveillance and management strategies for patients with LS and those with pMMRd only. 

## 2. Methods

### 2.1. Study Cohort and Data Collection

Between July 2018 and January 2021, we prospectively enrolled 451 cancer patients at the Koo Foundation Sun Yat-Sen Cancer Center (KF-SYSCC) in Taiwan. Participants were suspected of having Lynch Syndrome by clinical criteria based on the revised Bethesda guidelines [20]. The eligibility criteria included adults who had either colorectal cancer or endometrial cancer diagnosed before age 50, a significant family history of LS-related cancers, a personal history of two or more LS-related cancers, or colorectal or endometrial tumors with MMR protein deficiency by immunohistochemistry (IHC). All participants were unaware of their mismatch repair gene variant status before enrollment. Clinical information was gathered from participant surveys, electronic health records, and the Taiwan Cancer Registry.

This study was conducted in accordance with the Declaration of Helsinki, and the study protocol was approved by the Institutional Review Board at the Koo Foundation Sun Yat-Sen Cancer Center (case No. 20180301A). Written informed consent was obtained from each study participant.

### 2.2. Genetic Testing for Cancer Susceptibility Genes

All enrolled patients underwent germline genetic testing using a customized panel of cancer susceptibility genes (Qiagen, Hilden, Germany, QIAseq DNA panel CDHS-15111Z-1792; genes listed in Table S2), which included the MMR genes *MLH1*, *MSH2*, *MSH6*, *PMS2*, and *EPCAM*. Service providers executed genomic DNA extraction, sequencing library preparation, quality checks, and sequencing using amplicon-based PCR-amplified sequencing on Illumina MiSeq [21]. Unique molecular identifier (UMI)-embedded sequence reads were processed with smCounter2 [22] bundled software for the gene panel for variant calling. Duplicate reads in smCounter2-produced binary alignment map (BAM) files were removed by UMI-tools (https://umi-tools.readthedocs.io/en/latest/index.html) before copy number variation analyses with Quandico [23], ONCOCNV [24], and CoNVaDING [25]. Variants were annotated with Ensembl Variant Effect Predictor (VEP) [26]. Pathogenicity for each annotated variant was classified by TAPES [27], which implements the ACMG/AMP guidelines [28]. Variants classified as pathogenic or likely pathogenic (P/LP) were further inspected with annotated information and alignment results. The germline variant classifications on the ClinVar database (https://www.ncbi.nlm.nih.gov/clinvar/) and the consensus InSiGHT variant classifications on the InSiGHT database (https://insight-database.org/) were referenced for classification if available. Germline MMR deficiency (gMMRd) was defined as applying only to pathogenic or likely pathogenic variant carriers.

### 2.3. Immunohistochemistry (IHC) for Tumor Samples

Immunohistochemistry for the MMR proteins MLH1, MSH2, MSH6, and PMS2 was performed using an automated staining instrument (BenchMark XT automated staining instrument, Ventana Medical System, Basel, Switzerland). Four-micrometer-thick sections were obtained from formalin-fixed, paraffin-embedded blocks of the selected tumor sections. The tissue sections were pretreated with heat-induced antigen retrieval in Tris-EDTA based buffer (pH 7.8) at 95 °C (Cell conditioning Solution, CC1, Ventana Medical System, Basel, Switzerland) for 56 to 64 min (MLH1—60 min, MSH2—56 min, MSH6—60 min, PMS2—64 min). Peroxidase activity was blocked for 5 min before incubation of the tissue sections with primary antibodies for 1 h at 37 °C. Primary antibodies applied for the IHC analyses were: MLH1 (1:50, clone GM011, Genemed Biotechnologies, San Francisco, CA, USA), MSH2 (1:50, clone G219-1129, Cell marque, Rocklin, CA, USA), MSH6 (1:200, clone EPR3945, Abcam, Cambridge, UK), and PMS2 (ready to use, clone EP51, Dako agilent, Tokyo, Japan). The antigen–antibody reaction was detected with anti-mouse/rabbit polyclonal—horseradish peroxidase polymer, with DAB as substrate. The detection kits were the ultraView Detection Kit (Ventana Medical System, Basel, Switzerland) for MLH1 and MSH6 and the OptiView Detection Kit (Ventana Medical System, Basel, Switzerland) for MSH2 and PMS2. Tissue sections were then counterstained with hematoxylin. The IHC results were interpreted by pathologists.

### 2.4. Clinical Correlation and Statistical Analyses

Colorectal or endometrial tumor characteristics, IHC stains when available, treatment modalities, and clinical outcomes were extracted from the institutional electronic health record, the Taiwan Cancer Registry, and participant surveys. Clinical characteristics between groups were analyzed using a Chi-squared test for categorical variables, and *t*-test (2 groups) or ANOVA (>2 groups) for continuous variables. 

Survival analyses were performed using Kaplan–Meier curves. The primary endpoint was relapse-free survival, defined as the time from cancer diagnosis to the first appearance of cancer relapse in a patient with disease previously in clinical remission, or death without known cancer relapse. The secondary endpoint was overall survival, defined as the time from cancer diagnosis to death from any cause. For patients who did not experience an endpoint event, the times were censored at the date of the last follow-up visit, or at the date at which the patient was last known to be alive for the analysis of overall survival. 

The primary and secondary endpoints of the patients with or without gMMRd were compared using Kaplan–Meier curves. Statistical significance between the survival curves was evaluated by the log-rank test. The Cox proportional hazards regression model could not be carried out due to zero relapse events in the gMMRd (+) group. Survival comparison was also carried out in the subgroups, including gender, age of cancer onset, cancer type/location, stage, chemotherapy, and radiotherapy. 

For the comparison among the gMMRd (+), gMMRd (−) pMMRd (+), and gMMRd (−) pMMRd (−) groups, survival analysis was conducted using Kaplan–Meier curves. Statistical significance was evaluated using the log-rank test. A post-hoc test comparing the survival between each pair was conducted using the log-rank test with Benjamini–Hochberg adjustment for multiple comparisons. A P-value less than 0.05 was defined as statistically significant for rejecting the null hypothesis. All analyses were performed using the R language. 

## 3. Results

### 3.1. Study Population and Germline MMR Pathogenic Variants (gMMRd)

The study cohort included 451 cancer patients clinically suspected of having Lynch syndrome by the revised Bethesda guidelines. All participants were of Chinese ethnicity, 65.4% were female, 13.1% had two or more cancers, and the median age of cancer onset was 43 (range from 23 to 75). Overall, 36 (8.0%) patients were found to carry a pathogenic MMR gene variant. Among the 334 patients with colorectal cancer, 25 (7.5%) carried a pathogenic MMR gene variant; among the 125 patients with endometrial cancer, 14 (11.2%) carried a pathogenic MMR gene variant (Table 1). Among the pathogenic variant carriers, 18 (50.0%) occurred in *MLH1*, 15 (41.7%) in *MSH2*, 2 (5.6%) in *MSH6*, and 1 (2.8%) in *PMS2* (Table S1). 

Tumor characteristics of those with and without gMMRd are compared in Table 1. None of the patients were aware of their MMR gene variant status at the time of cancer diagnosis, treatment, or surveillance. Those with gMMRd were significantly more likely to have more than one cancer (30.6% vs. 11.6%; *p* < 0.0001). Among patients with colorectal cancer, gMMRd (+) patients were more likely to have cancer located in the proximal colon (48.0% vs. 17.2%; *p* = 0.0002). Other characteristics, including the age of onset, cancer stage, and receipt of chemotherapy or radiotherapy, did not show any statistically significant differences between patients with or without gMMRd. Among patients with endometrial cancer, those carrying gMMRd had a significantly higher chance of having stage 2 or 3 cancer (42.8% vs. 17.1%) rather than stage 1 cancer (50.0% vs. 76.6%; *p* = 0.014). There was no statistically significant difference in the age of onset or receipt of chemotherapy or radiotherapy.

### 3.2. Survival Analysis in Colorectal and Endometrial Cancer by gMMRd

**Figure 1:**
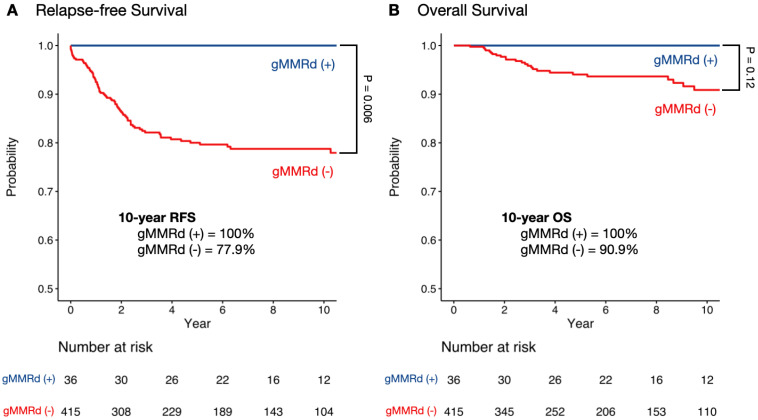
Relapse-free survival (**A**) and overall survival (**B**) in patients with colorectal cancer or endometrial cancer, comparing those with gMMRd (Lynch syndrome) and those without.

To evaluate the prognostic value of gMMRd for colorectal or endometrial cancer, we performed survival analyses on relapse-free survival (RFS) and overall survival (OS) between patients with or without gMMRd (Figure 1). After a median follow-up of 60.8 months, 0 patients in the gMMRd (+) group had relapse or died, while 83 patients (20%) in the group without gMMRd had relapse or died. The rate of relapse-free survival at 10 years was 100% among gMMRd carriers, as compared with 77.9% among non-carriers (*p* = 0.006, Figure 1A). At 10 years, the overall survival in the patients with gMMRd was 100%, as compared with 90.9% in the patients without gMMRd (*p* = 0.12, Figure 1B).

Subgroup analysis based on patient and tumor characteristics (Table 2) revealed significantly better relapse-free survival for gMMRd (+) patients compared to gMMRd (−) patients in the following subgroups: male gender, age of cancer onset less than 50 years, colorectal cancer diagnosis, stage IV cancer, having received chemotherapy, and not receiving radiotherapy.

### 3.3. Characteristics and Survival for Colorectal and Endometrial Cancer Patients Based on gMMRd and pMMRd Status

From the original cohort, we retrieved all available IHC staining results for pMMRd status of the tumor samples from 102 patients without gMMRd. We found that 14 (13.7%) of them had pMMRd (+) (Table 3). Comparison of the gMMRd (+), gMMRd (−) pMMRd (+), and gMMRd (−) pMMRd (−) groups showed that the gMMRd (−) pMMRd (+) patients were significantly older in age of onset than the other two groups. Other characteristics, including tumor stage, receipt of chemotherapy or radiotherapy, and cancer type did not show statistically significant differences. 

**Figure 2:**
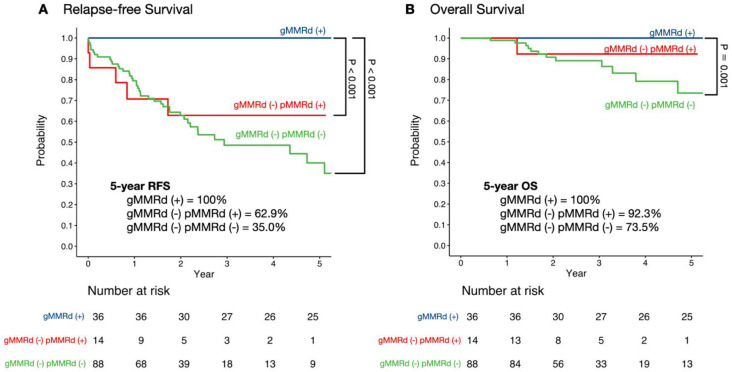
Relapse-free survival (**A**) and overall survival (**B**) in patients with colorectal cancer or endometrial cancer, comparing the presence or absence of germline MMR deficiency (gMMRd) and pathological MMR deficiency (pMMRd).

In the survival analysis, 5-year relapse-free survival rates were 100% for gMMRd (+), 62.9% for gMMRd (−) pMMRd (+), and 35.0% for gMMRd (−) pMMRd (−) patients (Figure 2A). Log-rank tests adjusted for multiple comparisons found significant differences between gMMRd (+) and gMMRd (−) pMMRd (+) groups and between gMMRd (+) and gMMRd (−) pMMRd (−) groups. The 5-year overall survival rates were 100% for gMMRd (+), 92.3% for gMMRd (−) pMMRd (+), and 73.5% for gMMRd (−) pMMRd (−) patients (Figure 2B). Log-rank tests adjusted for multiple comparisons revealed a significant difference in overall survival only between gMMRd (+) and gMMRd (−) pMMRd (−) groups. 

## 4. Discussion

In this cohort study, we demonstrated the long-term relapse-free and overall survival outcomes for patients with different gMMRd and pMMRd statuses. Patients with gMMRd had significantly better relapse-free survival than those without gMMRd, regardless of the pMMRd status (Figure 1A and Figure 2A). For overall survival, gMMRd (+) patients had a favorable outcome compared to those with gMMRd (−) pMMRd (−) (Figure 2B). Although the 100% relapse-free and overall survival rates seemed surprising in our study, previous studies have also shown good survival for patients with gMMRd (i.e., Lynch syndrome). A European database study of 1942 gMMRd carriers demonstrated similarly excellent 10-year crude survival rates: 87% after any cancer, 91% if the first cancer was colorectal, and 98% for endometrial cancer [9]. Another study in a Chinese cohort reported a 5-year overall survival rate of 97.6% and recurrence-free survival of 78% for Lynch syndrome patients [16]. Our results support the existing literature, suggesting favorable survival outcomes up to ten years for Chinese patients with colorectal or endometrial cancer. Moreover, we observed significantly better relapse-free survival for patients with Lynch syndrome compared to those without gMMRd in specific subgroups (Table 2) such as male gender, age of onset less than 50, those with colorectal cancer, stage IV cancer, and those who had received chemotherapy. Due to the absence of recurrence or death events in our gMMRd (+) group, we were unable to analyze potential differences in prognosis based on specific MMR genes. However, an international prospective study of 8500 gMMRd carriers with 71,713 follow-up years found no significant survival differences by specific genes after colorectal or endometrial cancer [29]. 

With advancements in tumor molecular analysis, the gMMRd (−) pMMRd (+) population has gained increased research interest. Several mechanisms were proposed for pMMRd without gMMRd, including hypermethylation of the *MLH1* promoter (considered a variant of Lynch syndrome), BRAF V600E somatic mutation (considered sporadic cancer), and Lynch-like syndrome if the previous two are absent [18,19]. Previous studies have shown a worse prognosis for patients with *MLH1* promoter hypermethylation or BRAF V600E mutation compared to those with gMMRd [8,13,30]. On the other hand, the prognosis of Lynch-like syndrome remains unclear due to its low prevalence and diverse hypotheses regarding pathogenesis [19,31]. Despite the heterogenous makeup of the gMMRd (−) pMMRd (+) population, our results (Figure 2) showed that these patients had intermediate (statistically insignificant) relapse-free and overall survival compared to those with Lynch syndrome or gMMRd (−) pMMRd (−). The clinical implication of this finding is that when the pathological analysis of a resected tumor reveals pMMRd, germline MMR genetic analysis may be crucial for prognosis, as gMMRd is associated with a significantly better prognosis compared to pMMRd. A recent Japanese cohort study found no significant difference in clinicopathological features between Lynch syndrome and Lynch-like syndrome, further emphasizing the importance of germline genetic diagnosis following the detection of pMMRd [32]. 

The strengths of this study include the long follow-up duration and the comprehensive genome sequencing for patients clinically suspected of LS. We also demonstrated comparable survival outcomes in the understudied Asian population compared to those reported in Western studies [9,19,31]. While this is a single-institution study, our investigation benefitted from the institution’s standardized cancer treatment and surveillance protocols, ensuring consistency in patient care and outcome assessments across the cohort. Our study has several limitations. First, data on *MLH1* promoter hypermethylation and BRAF V600E mutation, which could help elucidate the underlying causes for some of the gMMRd (−) pMMRd (+) patients, were unavailable. Second, the gMMRd (−) pMMRd (+) group lacked sufficient patients to achieve statistically significant survival differences. Implementing routine MMR IHC or MSI analyses for all at risk patients would enhance data available for future survival analysis. In addition, we lacked adequate data on newer tumor analyses (e.g., tumor mutation burden, tumor infiltrating lymphocytes) or more recent treatment modalities (e.g., immune check point inhibitors) to assess their impact on outcomes. These limitations highlight the need for further research in this area.

## 5. Conclusions

Our cohort study of Chinese cancer patients showed a favorable relapse-free and overall survival for patients with LS and an intermediate (statistically insignificant) outcome for patients with gMMRd (−) pMMRd (+) compared to those with gMMRd (−) pMMRd (−). These insights may pave the way toward a more precise approach to cancer treatment and surveillance strategies for these patients. 

## Figures and Tables

**Table 1:** Characteristics of colorectal and endometrial cancer with or without gMMRd, *n* = 451.

	gMMRd (−) *n* = 415	gMMRd (+) *n* = 36	*p*-Value
Female, *n* (%)	272 (65.5%)	23 (63.9%)	0.84
Number of cancers			<0.0001
1	367 (88.4%)	25 (69.4%)	
2	45 (10.8%)	8 (22.2%)	
≥3	3 (0.7%)	3 (8.3%)	
**Colorectal cancer**	***n* = 309**	***n* = 25**	
Age of onset, mean (SD)	42.4 (5.3)	42.7 (7.1)	0.81
Stage			0.22
0	9 (2.9%)	2 (8.0%)	
1	49 (15.9%)	2 (8.0%)	
2	81 (26.2%)	9 (36.0%)	
3	125 (40.5%)	8 (32.0%)	
4	43 (13.9%)	3 (12.0%)	
Unknown	2 (0.7%)	1 (4.0%)	
Location in colon			0.0002
Proximal *	53 (17.2%)	12 (48.0%)	
Distal *	255 (82.8%)	13 (52.0%)	
Chemotherapy	192 (62.1%)	14 (56.0%)	0.54
5-FU based	188 (97.9%)	14 (100.0%)	0.59
Radiotherapy	63 (20.4%)	3 (12.0%)	0.31
**Endometrial cancer**	***n* = 111**	***n* = 14**	
Age of onset, mean (SD)	43.2 (7.1)	44.5 (8.1)	0.53
Stage			0.014
1	85 (76.6%)	7 (50.0%)	
2	3 (2.7%)	3 (21.4%)	
3	16 (14.4%)	3 (21.4%)	
4	7 (6.3%)	1 (7.1%)	
Chemotherapy	25 (22.5%)	5 (35.7%)	0.28
Platinum-based	25 (100.0%)	5 (100.0%)	
Radiotherapy	26 (23.4%)	5 (35.7%)	0.32

* Proximal colon: cecum, ascending colon, hepatic flexure, transverse colon, and splenic flexure; distal colon: descending colon, sigmoid colon, and rectum.

**Table 2:** Subgroup analysis comparing relapse-free survival in those with or without gMMRd.

		Patients	Events	10-Year RFS (%)	*p*-Value
**Gender**					
Female	gMMRd (+)	23	0	100	0.063
	gMMRd (−)	272	39	83.9	
Male	gMMRd (+)	13	0	100	0.032
	gMMRd (−)	143	40	68.1	
**Age of onset**					
<50	gMMRd (+)	31	0	100	0.0099
	gMMRd (−)	499	74	74.0	
≥50	gMMRd (+)	5	0	100	0.19
	gMMRd (−)	16	5	67.5	
**Cancer type**					
Colorectal	gMMRd (+)	24	0	100	0.01
	gMMRd (−)	304	72	73.3	
Endometrial	gMMRd (+)	12	0	100	0.38
	gMMRd (−)	111	7	92.0	
**Cancer stage**					
Stage I	gMMRd (+)	9	0	100	0.45
	gMMRd (−)	134	8	92.2	
Stage II	gMMRd (+)	10	0	100	0.26
	gMMRd (−)	82	9	86.8	
Stage III	gMMRd (+)	11	0	100	0.12
	gMMRd (−)	140	29	76.0	
Stage IV	gMMRd (+)	4	0	100	0.032
	gMMRd (−)	49	32	30.6	
**Treatment**					
Chemotherapy	gMMRd (+)	18	0	100	0.026
	gMMRd (−)	207	49	74.4	
No chemotherapy	gMMRd (+)	18	0	100	0.094
	gMMRd (−)	208	30	81.5	
Radiotherapy	gMMRd (+)	9	0	100	0.18
	gMMRd (−)	78	16	77.8	
No radiotherapy	gMMRd (+)	27	0	100	0.016
	gMMRd (−)	337	63	77.9	
**Location of colon**					
Distal	gMMRd (+)	13	0	100	0.057
	gMMRd (−)	255	64	71.7	
Proximal	gMMRd (+)	12	0	100	0.12
	gMMRd (−)	53	9	81	

**Table 3:** Characteristics of patients based on gMMRd or pMMRd status, *n* = 138.

	gMMRd (+)	gMMRd(−) pMMRd (+)	gMMRd(−) pMMRd (−)	*p*-Value
*n*	36	14	88	
Age of onset, mean (SD)	43.6 (7.0)	53.2 (13.7)	44.1 (6.6)	0.0002
Stage				0.3257
0	2 (5.6%)	0 (0%)	1 (1.1%)	
1	9 (25.0%)	2 (14.3%)	13 (14.8%)	
2	10 (27.8%)	5 (35.7%)	19 (21.6%)	
3	11 (30.6%)	4 (28.6%)	29 (33.0%)	
4	4 (11.1%)	3 (21.4%)	26 (29.5%)	
Chemotherapy	18 (50.0%)	9 (64.2%)	42 (47.7%)	0.5156
Radiotherapy	9 (25.0%)	4 (28.6%)	11 (12.5%)	0.1266
Cancer type				0.1698
Endometrial cancer	12 (33.3%)	4 (28.6%)	16 (18.2%)	
Colorectal cancer	24 (66.7%)	10 (71.4%)	72 (81.8%)	

## Data Availability

The datasets used and/or analyzed during the current study are available from the corresponding author on reasonable request.

## Supplementary Materials

The following supporting information can be downloaded at: https://www.mdpi.com/article/10.3390/cancers16132342/s1, Table S1: Germline Mismatch Repair Gene Pathogenic Variants; Table S2: QIAseq DNA panel CDHS-15111Z-1792: gene summary.

## List of Abbreviations

MMR, Mismatch Repair; MMRd, Mismatch Repair Deficiency; RFS, Relapse-Free Survival; OS, Overall Survival; LS, Lynch Syndrome; CRC, Colorectal Cancer; EC, Endometrial Cancer; IHC, Immunohistochemistry; MSI, Microsatellite Instability; ANOVA, Analysis of Variance; SD, Standard Deviation.
