# Supplementary material for: Mismatch Repair (MMR) Gene Mutation Carriers Have Favorable Outcome in Colorectal and Endometrial Cancer: A Prospective Cohort Study
Source: Cancers (Basel). 2024 Jun 26;16(13):2342. doi: 10.3390/cancers16132342 (PMC11240388; doi:10.3390/cancers16132342)
Supplement: Supplementary file 1 [file cancers-16-02342-s001.zip › cancers-3038028-supplementary.pdf]

Supplemental Tables

Mismatch Repair (MMR) Gene Mutation Carriers Have Favorable Outcome in Colorectal and Endometrial Cancer: A Prospective Cohort Study

Table S1. Germline Mismatch Repair Gene Pathogenic Variants, N=36

Gene	Transcript	HGVSc	HGVSp	Variant type	SNP ID	#Carrier	MMR tumor IHC staining
MLH1	NM_000249.4	c.67del	p.E23fs	Frameshift Del	rs63750822	1	
MLH1	NM_000249.4	c.104_105insAA	p.M35fs	Frameshift Ins	rs587778882	3	
MLH1	NM_000249.4	c.199G>T	p.G67W	Missense	rs63750206	1	
MLH1	NM_000249.4	c.298C>T	p.R100*	Nonsense	rs63751221	1	
MLH1	NM_000249.4	c.461del	p.D154fs	Frameshift Del	novel	1	MLH1 loss, PMS2 loss
MLH1	NM_000249.4	c.640del	p.D214fs	Frameshift Del	rs1553644194	1	
MLH1	NM_000249.4	c.677G>A	p.R226Q	Missense	rs63751711	3	MLH1 loss, PMS2 loss (x2)
MLH1	NM_000249.4	c.793C>T	p.R265C	Missense	rs63751194	3	MLH1 loss, PMS2 aberrant loss (x1)
MLH1	NM_000249.4	c.1489dup	p.R497fs	Frameshift Ins	rs63750855	1	
MLH1	NM_000249.4	c.1989+1G>T	-	Splice Site	rs267607879	1	
MLH1	NM_000249.4	c.2041G>A	p.A681T	Missense	rs63750217	2	
MSH2	NM_000251.3	c.942+2T>A	-	Splice Site	rs587779195	1	
MSH2	NM_000251.3	c.942+3A>T	-	Splice Region	rs193922376	2	MSH2 loss, MSH6 loss (x1)<br>MSH2 weak, MSH6 loss, MSI-H (x1)
MSH2	NM_000251.3	c.943-1G>A	-	Splice Site	rs12476364	1	MSH2 loss, MSH6 loss
MSH2	NM_000251.3	c.1216C>T	p.R406*	Nonsense	rs63751108	1	
MSH2	NM_000251.3	c.1226_1227del	p.Q409fs	Frameshift Del	rs63750086	3	MSH2 loss, MSH6 loss (x1)
MSH2	NM_000251.3	c.1440dup	p.L481fs	Frameshift Ins	rs1666728055	1	MSH2 loss, MSH6 aberrant loss
MSH2	NM_000251.3	c.1857T>G	p.Y619*	Nonsense	rs63750312	1	
MSH2	NM_000251.3	c.1861C>T	p.R621*	Nonsense	rs63750508	1	
MSH2	NM_000251.3	c.2087C>T	p.P696L	Missense	rs267607994	1	MSH2 loss, MSH6 loss
MSH2	NM_000251.3	c.2131C>T	p.R711*	Nonsense	rs63750636	1	MSH2 loss, MSH6 loss
EPCAM /MSH2	NM_002354.3 /NM_000251.3	del exon 1-9 /del exon 1-7	-	LGR	novel	2	MSH2 loss, MSH6 loss (x1)
MSH6	NM_000179.3	c.2731C>T	p.R911*	Nonsense	rs63751017	1	
MSH6	NM_000179.3	c.2823dup	p.A942fs	Frameshift Ins	novel	1	
PMS2	NM_000535.7	del exon 9-11	-	LGR	novel	1	

LGR: large genomic rearrangement

Table S2. QIAseq DNA panel CDHS-15111Z-1792: gene summary

Gene	No. of base pairs in the region of interest (ROI)	No. of base pairs not covered by fragments <= 150 bp
APC	8857	0
ATM	9791	0
BARD1	2444	0
BMPR1A	1709	0
BRCA1	5888	0
BRCA2	10517	0
BRIP1	3940	0
CDH1	2809	0
CHEK2	1911	0
EPCAM	1035	0
GREM1	743	0
MET	4494	0
MLH1	2461	0
MRE11A	2356	0
MSH2	3107	0
MSH3	3654	0
MSH6	4183	0
MUTYH	1810	0
NBN	2425	0
NF1	9300	0
PALB2	3691	0
PMS1	2972	0
PMS2	2739	258
POLD1	3662	0
POLE	7351	0
PTEN	1302	0
RAD50	4189	0
RAD51C	1225	0
RAD51D	1276	0
RECQL	2090	37
RINT1	2529	0
SLX4	5645	0
SMAD4	1769	0
SMARCA4	5411	0
STK11	1408	0
TP53	1383	0
XRCC2	873	0
